# Supplementary material for: Distribution, dynamics, and physiological races of wheat stem rust (Puccinia graminis f.sp. tritici) on irrigated wheat in the Awash River Basin of Ethiopia
Source: PLoS One. 2021 Sep 23;16(9):e0249507. doi: 10.1371/journal.pone.0249507 (PMC8459957; doi:10.1371/journal.pone.0249507)
Supplement: S1 Table — (DOCX) [file pone.0249507.s001.docx]

# Supporting File - 1

S1 Table. Irrigated wheat varieties were affected by the *Pgt* races identified in 2018/19 and 2019/20 cool seasons and their frequency from the total viable *Pgt* population in the areas.

| **Races** | **Varieties/lines affected** | **Freq. of the race in 2018/20** | **Freq. of the race in 2019/20** |
| --- | --- | --- | --- |
| **TTTTF** | Danda’a, Fentale-2, Kakaba, Gaambo | 1(25) | 15(45.5) |
| **TKTTF** | Kakaba, Fentale-2, Danda’a | 1(25) | 5(15.2) |
| **TKKTF** | Kakaba, Fentale-2, | 1(25) | 8(24.2) |
| **TTKTF** | Kakaba, Danda'a, | 1(25) | 4(12.1) |
| **TKPTF** | Unknown | 0 | 1(3.0) |
| **Total** |  | 4 | 33 |

The figure in the parenthesis indicated the frequency (%) of a specific race in the season.

Irrigated wheat crop production is grown in the cool crop growing season with improved crop varieties in the Awash River basin of Ethiopia. In the Awash River Basin, wheat production has been increasing to substantial levels to support wheat self-sufficiency. On the other hand, stem rust disease prevalence, incidence, and severity have been increasing, and the disease may reach a level that threatens wheat production and productivity. Therefore, it is essential to take appropriate action to avert a disaster from occurring.

The wheat production trend increases at an alarming rate with the intention of the government of Ethiopia to substitute the imports and stabilize the wheat self-sufficiency. Thus, a plan could become possible by deploying best-bet wheat production technologies (MOA, 2020). Wheat production and expansion to potential irrigated areas of Ethiopia was fast progress. The country also has a huge potential and workable plan to cultivate the different basins by using the water resources in the existing irrigable lands of the country. Among the high potential areas, the Abay River Basin, commonly known as the Upper Nile River region and Awash River Basin are the major ones. The areas are located, in the northwest highlands and western foothill lowlands of Ethiopia and the Central rift v the basin. Our little experience, for the past six years both in area expansion and cropping frequency, one of the potential problem or challenge which needs a strategic intervention were biotic stress majorly stem rust.
