# Supplementary material for: Distribution, dynamics, and physiological races of wheat stem rust (Puccinia graminis f.sp. tritici) on irrigated wheat in the Awash River Basin of Ethiopia
Source: PLoS One. 2021 Sep 23;16(9):e0249507. doi: 10.1371/journal.pone.0249507 (PMC8459957; doi:10.1371/journal.pone.0249507)
Supplement: S1 Fig — (DOCX) [file pone.0249507.s002.docx]

# Supporting File - 2


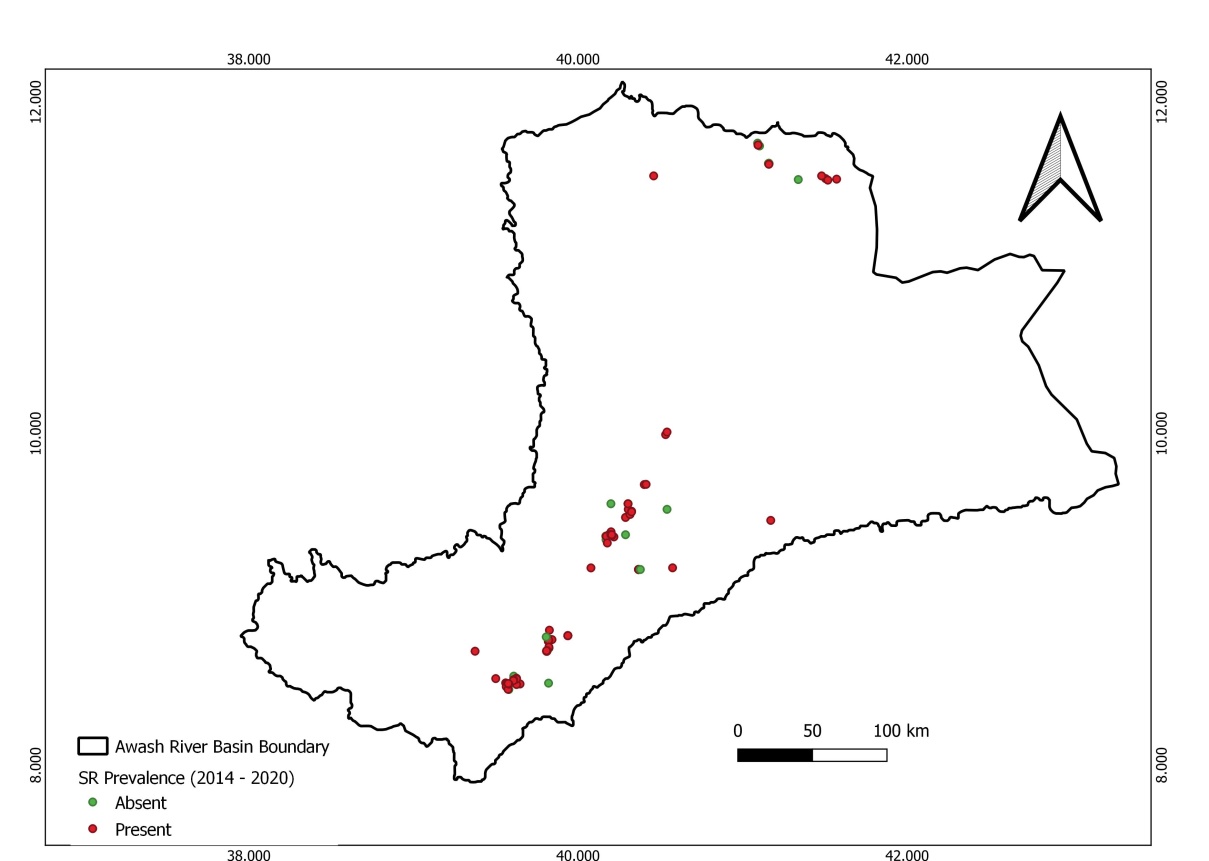


**S1** Fig. The wheat survey plot maps

<a rel="license" href="http://creativecommons.org/licenses/by/4.0/"><img alt="Creative Commons License" style="border-width:0" src="https://i.creativecommons.org/l/by/4.0/88x31.png" /></a><br />This work is licensed under a <a rel="license" href="http://creativecommons.org/licenses/by/4.0/">Creative Commons Attribution 4.0 International License</a>.
