# Supplementary material for: Distribution, dynamics, and physiological races of wheat stem rust (Puccinia graminis f.sp. tritici) on irrigated wheat in the Awash River Basin of Ethiopia
Source: PLoS One. 2021 Sep 23;16(9):e0249507. doi: 10.1371/journal.pone.0249507 (PMC8459957; doi:10.1371/journal.pone.0249507)
Supplement: S1 Text — (DOCX) [file pone.0249507.s003.docx]

# Supporting File - 3

S1 Text. *Pg*t Genetic diversity in upper Awash River basin of Ethiopia; core SNP assay CDL results from Minnesota
