# Supplementary figures and images for: Distribution, dynamics, and physiological races of wheat stem rust (Puccinia graminis f.sp. tritici) on irrigated wheat in the Awash River Basin of Ethiopia
Source: PLoS One. 2021 Sep 23;16(9):e0249507. doi: 10.1371/journal.pone.0249507 (PMC8459957; doi:10.1371/journal.pone.0249507)

# Supporting File - 4

S2 Text. Irrigated wheat field in West Arsi zone, Jeju districts, mid lands.


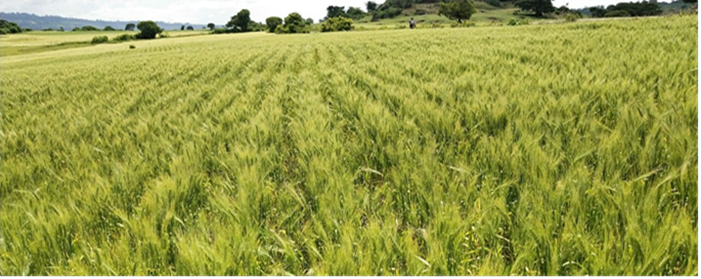

Supplement: S2 Text — (DOCX) [file pone.0249507.s004.docx]
