# Supplementary material for: Distribution, dynamics, and physiological races of wheat stem rust (Puccinia graminis f.sp. tritici) on irrigated wheat in the Awash River Basin of Ethiopia
Source: PLoS One. 2021 Sep 23;16(9):e0249507. doi: 10.1371/journal.pone.0249507 (PMC8459957; doi:10.1371/journal.pone.0249507)
Supplement: Attachment — Submitted filename: Response to Reviewers.docx [file pone.0249507.s006.docx]

**Response to Reviewers**

First of all, I would like to thank for the academic editor and reviewers of **PLOS ONE** journals for the nice attentive editing and comments they forwarded with in this immature manuscripts, it helps a lot for its improvements. Please present my gratitude for them with warm gratitude with ‘thank you very much!”

Saying this almost all of the comments given with the resource persons are acceptable and accepted and helps the improvement of the manuscript with the following manner. The author is not native English speaker, so there could be limitations in understanding what the editors and reviewers want to see on effect but positive to change and learn from them. Most of the reviewers’ comments are addressed.

Reviewer #1: Comments to authors

This paper reports a study conducted on ‘distribution, dynamics and physiological races of Pgt of wheat grown in irrigated areas of the Rift Valley region of Ethiopia’. The paper utilizes survey results of the stem rust prevalence, incidence and severity collected for five seasons. Additionally race virulence studies conducted in the lab over two seasons. I have no concern with trial set up – either for the field studies or lab work, however I have a general question whether this paper fits to PONE journal unless authors will make critical revisions and improvement in their writing. Authors used many Tables some of which can be combined and referred in the text rather. However, authors fail to refer Tables and Figures in the paper (Please below). The discussion could be improved better by discussing the research findings and removing some irrelevant topics. Please see some of my specific comments as below.

We try to improve the article

Line#14: Title ‘Epidemics’ should be written in lowercase letter as ‘epidemics’ for uniformity. Were there Pgt epidemics that happened during this study? Why are authors using this word in the title? Accepted but I used this, because there were no farms which is not sprayed to control the Pgt in 2018/19 and 2019/20 production years

Abstract

Line#17: I suggest using either ‘high value’ or ‘important’ or ‘major crops’. Authors are repeating similar terms. Would this be ok? ‘Wheat (Triticum aestivum L.) is one of the highly valued cereal crops in the world.’ Accepted

Line#23: ‘… and healthy plants were scored…’. How are healthy plants scored? This seems impractical and the sentence itself is vague, please reword it.

Line#26: There was no mention of location names for ‘…the two regions.’ Which regions are they representing? How about the ‘…six districts of Awash River basin’ mentioned in lines#21-22? Please make this clear. We described in clear manner in the methodology

Line#30: ‘…which, however,’ this sentence is wordy. Please remove ‘which,..’ accepted

Line#34: Do you mean ‘These races are…?’ please replace thus with these. accepted

Line#35: where is the evidence for this statement? TTTTF and TKKTF are the widest virulence spectrum which affects 90% of the Sr genes. Any previous research report? There was reports but it is in our observation and data two referred in S 1 table.

ETHICAL STATEMENT

Please rewrite this: ‘Still, we give maximum care during surveying through spore-free through self-sanitation after Pgt infested field observation to minimize induced disease dissemination to the communities in the production areas that no specific permissions were required for these locations.’ It is wordy and not clear. Accepted, what we did is that during surveying the after diseased field observation we try to sanitize our trousers and hands.

Introduction

your introduction needs re-organization and condensation. For example, Paragraph 2 (lines 65-81) that presents about irrigated wheat production in Ethiopia and associated biotic and abiotic problems can be combined with paragraph 4 (lines 94-104) and shortened. Please see other paragraphs too. Accepted and impacted

Lines 55-59: The sentences are wordy and hard to follow. Please see if this is correct and replace. ‘As the world population is expected to reach nine billion by 2050 (Edmeades et al., 2010), there should be an urgent need to increase the productivity of crops like wheat to meet the increasing demand for food (Weigand, 2011). In sub-Sahara Africa (SSA) wheat is grown on a total area of 2.9 M ha with an annual production amounting 7.5 MT (FAO, 2017).’ Accepted

Line#90: Leppik, (1970). Remove the comma (,) next to Leppik. Would it be ok to use more recent literatures? There is a lot of work on stem rust in East Africa following the emergence of Ug99. See literatures below and many more: I removed Leppik it was too old which doesn’t describe the present scenario and reviewed these recommended journals to validation of results

Lines 120-121: Please be specific. Is the in outbreak of 2013 in Ethiopia? “A huge Pgt outbreak by race TKTTF in 2013 caused up to 100% yield losses in some fields (Sanders, 2011). Replaced with Wurku etal,18 and Hie etal,2016

Line 122: the sentence is incomplete. Please rewrite.

Line 124: …national scale economic loss… this is not in your objective. Please be specific.

Line 125: …race genetic… ? I don’t understand what this means, better be replaced by …genetic analysis of isolated Pgt races in Cereals Disease Lab (CDL) at University of Minnesota … if it is ok. Yes

Line 127: please make sure italicizing *Pgt* throughout the paper. done

Lines 132-133: This needs rewording. Is this ok? The objective of this study was to determine the diseases intensity distribution, epidemics dynamics, in the irrigated belts of Awash River basin and to analyze the virulence of isolated Pgt races on seedling plants. *Accepted*

Materials and Methods

Line 145: Under sub-title ‘Survey of Wheat Stem Rust Distribution in Awash River Basin Fields’ authors did not indicate the number of fields surveyed by location and years 2014/15 to 2019/20. It is too general and unclear. I suggest authors to show how many fields in each district were visited for each growing season. We described

Line 159: How was ‘physiological races virulence spectrum’ assessment made? Please explain this. It is explained and easily identified in the supporting S1 Table. Any way the virulence spectrum of a race can be screened and magnified using the 20 Sr genes.

Lines 172-174: ‘Five seedlings of this variety …’ it is not clear which wheat variety was used. McNair? Yes

Line 236: Table 3 was not referred in the text. referred

Results

Lines 253-255: Was it only Gambo variety planted to all irrigated areas in 2016? Re-word this statement - “This means the diseases stem rust was not economically important in those seasons on wheat variety 'Gambo'. The next sentence is also self-contradictory to me. If stem rust was there before 2016, how could it be new in the year 2016?

This is not the case, even though stem rust was detected in 2014 and 2015 production years we couldn’t detected in 2016. Any way we try to impact it.

Lines 257-259: Is this ok? “In 2018/19, a total of 33 stem rust live samples were collected and 12.12% the samples were viable, whereas in 2019/20 a total of 46 samples were collected out of which 71.74% were viable.”

Huge variation, It is not ok even for me as a researcher but what else we can do? rather than doting down what we find and discussed it in the discussion.

Line 260: ‘… for the two seasons, 46.84% of them were viable.’ Values placed in BRACKETS should be out. The next phrase and sentences should be in the discussion section. Please delete this and consider discussing later: which are low overall samples were viable which is low overall accepted

261 viability could be associated with loss of sample viability with storage environmental condition. The 262 low viability of samples could be attributed to the late arrival of the collected samples or sample miss 263 handling….

Lines 263-266: I can see a lack of uniformity in writing. No need to write all the values (numbers) in the text. If necessary, please use proportions rather than quantity for the ease of understanding. The details can be summarize in a Table and referred in the write up. Would this be ok? “The spatial viability the stem rust samples collected in 2019/20 in Oromia Regional State was 40% in Sire, 100% in Jeju and 70.59% in Fentale districts, while in Afar Regional State the viability was 33.3% in Afambo, 100% in Amibara and 25% in Dubti districts. Accepted

Line 275: “This could be attributed to sunny weather 15 December -15 January.” This statement is wrong. How can sunny weather increases stem rust pressure by itself. There could be several factors in the disease triangle attached to it. This was not to say like that the sunny weather free of clouds resulted in disease free season.

Line 279: Figure 2 was not referred in the paper Refered.

Line 304: Table 3 was not referred in the paper. Line 311: Table 4 was referred once but it was missing where it should be referred. Line 318: Table 5 was not referred in the paper. Line 320: Table 6 was not referred in the paper. Line 324: Table 7 was not referred in the paper. Line 324: Table 8 was not referred in the paper. Line 401: Table 9 was not referred in the paper. Line 406: Table 10 was not referred in the paper. Line 410: Table 11 was not referred in the paper. Line 418: Table 12 was not referred in the paper. We combined and referred the table

Line 335: …in Figure 3A… Line 346…Figure 3B

Discussion

Line 439: …’wheat’ use lowercase letter.

Lines 439-444: The first two paragraphs are very broad or not relevant to this study. Removed

The discussion section needs further improvement if this work is going to be published on PONE. Acted

Line 643: Appendix Table 1 was not referred in the paper. (removed as a supporting file)

Reviewer #2: 1. General

- In general the manuscript was well prepared. As such findings are very important to get to the public in regard to planning approaches at developing rust resistant germplasms; it would be of great value to reflect what distinguishes this work from others, and what new findings are evident, and how might this work be used for strategies in developing better germplasm. It is also important that the statements made are as accurate as possible due to the importance of the work, I have described my comments as follows: Thank you Very much

- Title is long

- Some areas need English editing we try to improve it

- Italicize gene names, eg. Page 5 (ln 223 -225), Page 6 (table 2), many places in the text done

- Avoid starting sentences with acronyms	accepted Impacted

2. Abstract:

- Comments are shown on the attached document all are accepted

3. Introduction

- Too much detail about wheat production in Ethiopia. Instead better if you focus on the background of the problem we reduced

- It is stated as one objective but that is not the case (Page 2, ln 132-133) seen and improved with the first reviewers suggestions

4. Materials and methods

- What percentage of incidence and/or severity rate is a considered as a cut score to classify a field as diseased during the survey? For management 5% incidence but as a science who want to study in the disease dynamics score even for trace

- Figure 1 looks distorted (replaced)

- At which growth stage of the plant was the survey conducted? From booting to physiological maturity avoiding teliospores

5. Result

- Too many tables. Move tables that are less cited in the text put them as supplemental materials. Acted

6. Discussion

- Remove the first two paragraphs (lines 439-452) as they are less relevant. Instead, try to exploit similar works and compare with your results, ex. Meyer et al. 2021, Lemma et al. 2014, etc….(Reviewed and disscussed)

7. Conclusion

- Avoid vague conclusion (Page 19, lines 512 to 514) accepted
