# Supplementary material for: Distribution, dynamics, and physiological races of wheat stem rust (Puccinia graminis f.sp. tritici) on irrigated wheat in the Awash River Basin of Ethiopia
Source: PLoS One. 2021 Sep 23;16(9):e0249507. doi: 10.1371/journal.pone.0249507 (PMC8459957; doi:10.1371/journal.pone.0249507)
Supplement: Attachment — Submitted filename: Response to Reviewers.docx [file pone.0249507.s008.docx]

**Response to Reviewers (2)**

Once again, we would like to thank for the academic editor and reviewers of **PLOS ONE** journals for the nice attentive editing and comments they forwarded with in this manuscripts, it helps a lot for its improvements. Please present my gratitude for them with warm gratitude by ‘Thank you very much!”

Saying this almost all of the comments given with the resource persons are acceptable and accepted and helps the improvement of the manuscript with the following manner. The author is not native English speaker, so there could be limitations in understanding what the editors and reviewers want to see on effect but positive to change and learn from them. Most of the reviewers’ comments are addressed.

Journal Requirements:

Please review your reference list to ensure that it is complete and correct. If you have cited papers that have been retracted, please include the rationale for doing so in the manuscript text, or remove these references and replace them with relevant current references. Any changes to the reference list should be mentioned in the rebuttal letter that accompanies your revised manuscript. If you need to cite a retracted article, indicate the article’s retracted status in the References list and also include a citation and full reference for the retraction notice.

[Note: HTML markup is below. Please do not edit.]


6. Review Comments to the Author

Reviewer #1: Authors have made a lot of improvements over the previous draft. However, there still are some topographical errors requiring further formatting and proof reading. Below, please find a few examples:

Abstract
Line24: Sr-31 and Sr-24 could preferably written as Sr31 and Sr24. Please use similar naming in the rest of the paper. Accepted and I try to make similar naming in the rest of the paper
Introduction
Line110: Please change this citation to PLOS referencing system. Please see: Kumar et al. 2011 Accepted

Line 129. Author names should show up in full not as numbers at the start of any sentence. For example, [4,10] reported wheat …. should be modified as Tadesse et al. [4] and Rosegrant et al. [10]. Accepted

Line 141-142: In Ethiopia, hot spot areas for the appearance of virulent genetic diversity of stem rust
races were reported by [12]. … by Hailu et al. [12]. Please make modifications throughout the paper where applicable. Accepted

Materials and methods
Line187: Please delete 2.2. from sub-tile Survey of Wheat Stem Rust Distribution in Awash River Basin Fields Accepted \

Line188: The survey and surveillance assessment were assessed, among the farmers' fields at every 5-10 km ….could this be modified? My suggestion: The field assessments were conducted in the farmers’ fields at 5-10 km interval between wheat farms following the main road routes. Accepted

Line 194: Please replace Afambo&Asaita by Afambo and Asaita, otherwise it confuses readers. Accepted

Line210: via [22]… not clear. Please use author name followed by reference code. See above the comment. Acted

Line216: Data, on disease intensity like prevalence,…. the comma (,) after ‘Data’ is not required and could be modified as... Data on disease intensity like prevalence… Acted

Result
Line434-435: This statement doesn’t read well, please modify. Distribution and frequency of Pgt Races in Awash River basin for 2014/15 - 2017/18 off seasons in irrigated wheat cropping season there was no race analysis, I try to figure out it

Discussion
Line498-500: This sentence has something missing or not clear! …. Did authors meant to say: The stem rust survey conducted over the past six years in the irrigated wheat production in the Awash River Basin of Ethiopia indicated a trend of increase in the distribution, dynamics, and physiological races of wheat stem rust with time. Exactly

Line594: Make a brief conclusion if any. The subtitle ‘Conclusion’ is also not necessary and could be deleted. After all, this section is seemingly a summary of the research output than a conclusion. Paragraphs placed under the conclusion can be incorporated to the discussion section.

Usually scientific studies contain conclusions from the premises which are results so could it be accepted as summary and Conclusion. Still I acted on it to improve.

Reviewer #2: Thank you for addressing most of the previous comments. But the manuscript still needs some cleaning and editorial work. I have included all my comments in the attached pdf file. Some of the issues are:
- Inconsistency in using acronyms(Accepted)
- Formatting Ex. Italicize markers and genes(Accepted)
- Incomplete and unclear lengthy sentences(Accepted)
- Conclusion part is not presented(Acted)
- Unnecessary paragraph in the discussion part(Accepted)

7. PLOS authors have the option to publish the peer review history of their article ([what does this mean?](https://journals.plos.org/plosone/s/editorial-and-peer-review-process#loc-peer-review-history)). If published, this will include your full peer review and any attached files.

Yes for me!
